# Antioxidant, Antibacterial, and Bioaccessibility Properties of Ultrasound-Extracted Chilean Propolis

**DOI:** 10.3390/antiox14060651

**Published:** 2025-05-28

**Authors:** Jessica Mejía, Claudia Giovagnoli-Vicuña, Cristian Jacob, Gloria Montenegro, Andrea I. Moreno-Switt, Ady Giordano

**Affiliations:** 1 Departamento de Ciencias Vegetales, Facultad de Agronomía y Sistemas Naturales, Pontificia Universidad Católica de Chile, Santiago 7820436, Chile; jcmejia@uc.cl (J.M.); cjjacob@uc.cl (C.J.); gmonten@uc.cl (G.M.); 2 Departamento de Química Inorgánica, Facultad de Química y de Farmacia, Pontificia Universidad Católica de Chile, Santiago 7820436, Chile; 3 Escuela de Medicina Veterinaria, Facultad de Agronomía y Sistemas Naturales, Facultad de Ciencias Biológicas, Facultad de Medicina, Pontificia Universidad Católica de Chile, Santiago 7820436, Chile; andrea.moreno@uc.cl

**Keywords:** polyphenols, ultrasound-assisted extraction, bioactive properties, digestion

## Abstract

Propolis is a honeybee product with significant biological properties, strongly influenced by geographic and floral origin as well as extraction method. Ultrasound-assisted extraction (UAE) is an emerging alternative for propolis preparation. This study aimed to determine the optimal UAE conditions for total polyphenol content (TPC) and total flavonoid content (TFC) in Chilean propolis and compare its antioxidant and antimicrobial capacities with a conventional method. UAE was performed with varying ethanol concentrations (50–90%), temperatures (20–60 °C), and extraction times (10–50 min), keeping the solid/liquid ratio constant at 1:10 (*w*/*v*). The optimal ethanolic UAE extract (OE) was compared to the conventional ethanolic extract (CE) for antimicrobial activity against *Salmonella enterica* and *Listeria monocytogenes* and antioxidant capacity (DPPH, ABTS, FRAP assays). Optimal UAE conditions were 80% ethanol, 30 °C, and 30 min. The OE showed significantly higher (*p* < 0.05) TPC (22.4 ± 0.3 mg GAE/mL), TFC (15.7 ± 0.7 mg QE/mL), antioxidant capacity (ABTS: 35.7 ± 0.9; DPPH: 62.9 ± 0.3; FRAP: 49.7 ± 2.1 µmol TE/mL), and antimicrobial activity in the evaluated Gram-positive bacteria (>15 mm inhibition halo, MBC = 3.1 mg/mL) compared to the CE. The bioaccessibility analysis revealed that the OE maintained 20.1% of its polyphenol content and 69.5%, 60.5%, and 61.9% of DPPH, ABTS, and FRAP, respectively, after simulated gastrointestinal digestion. The established optimal UAE conditions generated extracts with increased polyphenol content, antioxidant capacity, antimicrobial activity, and bioaccessibility, indicating its potential as an extraction method for propolis with enhanced bioactivity.

## 1. Introduction

Propolis is produced by honeybees (*Apis mellifera*) from the exudates of the bark and various tissues of plants to control the growth of microorganisms [1]. This natural substance is characterized by a complex chemical composition [2], generally consisting of 50% resin, 30% wax, 10% essential oils, 5% pollen, and 5% other substances [3]. The chemical composition of propolis, and, consequently, its biological properties, varies substantially depending on multiple phytogeographic factors, such as vegetation, season, and environmental conditions [2,4].

Ultrasound-assisted extraction (UAE) is an effective extraction method based on acoustic cavitation, where ultrasonic waves generate microbubbles that collapse violently, producing high localized pressures and temperatures. This phenomenon induces shear forces, microstreaming, and microjetting, which promote matrix disruption and enhance solvent penetration and mass transfer [5,6]. By contrast, conventional extraction methods have lower selectivity for compounds of interest, low extraction yields, and long extraction periods (i.e., maceration for 28 days) [7]. Therefore, switching from a conventional extraction to an optimized UAE method would allow for obtaining extracts with a high proportion of their free bioactive compounds and greater biological properties such as antioxidant capacity and antimicrobial activity.

Chilean propolis is characterized by its botanical origin in native plant species such as *Lithraea caustica*, *Quillaja saponaria*, and *Baccharis linearis* [8,9]. These species are part of the evergreen matorral vegetation of central Chile, located in a Mediterranean semiarid region. These unique floral sources contribute to a distinctive chemical profile rich in phenolic compounds, flavonoids, and diterpenes, which varies significantly from that of propolis in other geographical origins [9,10]. Despite its promising biological potential, Chilean propolis remains underexplored in the global scientific literature, particularly in terms of its phytochemical diversity and functional applications.

Propolis has been widely recognized for its strong biological properties, largely attributed to its high content of phenolic compounds, which exhibit both antioxidant and antimicrobial activities [11,12]. These bioactive compounds play a key role in free radical-scavenging agents, thus reducing oxidative stress [13]. In addition, propolis can effectively inhibit the growth of microorganisms depending on the propolis’s geographical origin and the resistance of these microorganisms [1]. This makes propolis an important natural agent in food preservation and health-related applications [14]. Therefore, enhancing the extraction process can significantly increase the concentration of these bioactive compounds, thereby improving the extract’s overall biological properties, such as antioxidant capacity and antimicrobial effectiveness, and, consequently, its bioaccessibility.

Bioaccessibility refers to the fraction of the bioactive compound that is released from the food matrix to the gastrointestinal tract for luminal absorption [15]. To date, there have been in vitro models to evaluate how digestion acts on these compounds and, thus, predict their release from different food matrices. For this, simulations are carried out in different parts of the digestive system such as the oral cavity, stomach, small intestine, and large intestine [16].

The objective of this study was to optimize the extraction of propolis using UAE and compare the optimal ethanolic UAE extract (OE) with a conventional ethanolic extract (CE). Under optimal parameters, UAE exhibited greater antioxidant capacity, antibacterial activity, and bioaccessibility of bioactive compounds as compared to extracts obtained with a conventional method. These findings reveal the potential of UAE as an efficient technique for extracting Chilean propolis with enhanced bioactivity.

## 2. Materials and Methods

### 2.1. Raw Material and Sample Preparation

Propolis from *Apis mellifera* was acquired in 2023 from the central zone of Chile. The sample was stored at −20 °C until the extraction processes. The propolis sample had a moisture content of 2.0 ± 0.0 g/100 g, crude protein of 4.6 ± 0.3 g/100 g, total lipids of 56.8 ± 1.4 g/100 g, and crude ash of 1.3 ± 0.2 g/100 g. Analyses were performed according to AOAC methods [17].

### 2.2. Botanical Analysis of Propolis

For the palynological and plant structure analyses, 10 g of propolis samples was weighed and mixed with 20 mL of distilled water and stirred until a homogeneous dispersion was obtained. The mixture was then centrifuged at 1006× *g* for 10 min, and the supernatant was discarded. The sediment was resuspended in 20 mL of distilled water and vortexed to ensure uniformity. A 100 µL aliquot was taken and placed on a glass slide, and this process was repeated four times for consistency. A drop of Calberla’s fuchsin stain was added to facilitate the visualization of pollen grains under a light microscope. The identification and quantification of pollen grains and plant structures (trichomes, vessels, and epidermis) were performed by comparing them with reference materials, including specialized literature and the botanical bee pollen catalog at the Laboratory of Botany (Department of Plant Sciences, Faculty of Agronomy and Natural Systems, Pontificia Universidad Católica de Chile, Santiago, Chile). Additional comparisons were made using photographs and permanent preparations available at the same laboratory. The proportion of each structure in relation to the total counted structures was then estimated.

### 2.3. Ethanolic Extraction of Propolis

Conventional and ultrasound methods were used to obtain ethanolic extracts from the propolis sample. The conventional method for preparation of ethanolic propolis extract consisted of procedures routinely conducted by local beekeepers. Briefly, a mixture of 1 g of ground propolis and 10 mL of 70% (*v*/*v*) ethanol was placed in an amber Falcon tube and stored at room temperature for 28 days. During the initial 14 days, the mixture was manually shaken twice daily, followed by a 14-day undisturbed maceration period. Finally, the extract was centrifuged for 10 min at 1807× *g* (Bioprocen 22 R model, ORTO ALRESA, Madrid, Spain), and the resulting supernatant was recovered and stored at 4 °C for subsequent analysis.

To determine the OE, single-factor tests were conducted in an ultrasonic bath (Ultrasonic Cleaners, BIOBASE, Jinan, China; 80 W of power and a 40 kHz operating frequency). Three variables were evaluated: ethanol concentration (50–90%, *v*/*v*), temperature (20–60 °C), and extraction time (10–50 min). For each test, the remaining parameters were kept constant at 80% (*v*/*v*) ethanol, 25 °C, and 30 min. All experiments were conducted using a 1:10 (*w*/*v*) sample-to-solvent ratio, as described for the conventional extraction protocol. The ultrasonic power density was calculated by dividing the nominal power by the extract volume, yielding a value of 8 W/mL.

### 2.4. Phytochemical Screening

Ethanolic extracts of propolis were subjected to standard screening tests to determine the presence of phytochemicals. Secondary metabolites, such as tannins, flavonoids, coumarins, alkaloids, phenols, proteins, carbohydrates (sugars), and saponins, were quantified through procedures previously described [18] (Table 1). The relative concentrations of the compounds were determined using visual color intensity as an indicator compared to the positive control. +++ indicated high intensity and high concentration, ++ mild intensity and mild concentration, + low intensity and low concentration, and − indicated no change, with the compound not detected. The positive controls were tannic acid (1000 ppm), catechin (1000 ppm), coumarin (500 ppm), oxymatrine (800 ppm), gallic acid (1000 ppm), casein (10% *w*/*v*), sucrose (1% *w*/*v*), and oats (1% *w*/*v*). The negative control was distilled water.

### 2.5. Determination of TPC and TFC

The total polyphenol content (TPC) in the propolis extracts was determined using the Folin–Ciocalteu (FC) method described by Singleton et al. [19]. Propolis extracts (20 μL) were mixed with diluted FC reagent (100 μL at a 1:10 (*v*/*v*) ratio) and 7.5% sodium carbonate solution (80 μL). The spectrophotometric readings were taken at 765 nm after 40 min of dark incubation in a microplate reader (FlexA-200, ALLSHENG Instrument Co., Ltd., Hangzhou, China). Gallic acid was utilized to create a standard curve (50–400 μg/mL; y = 0.0026x + 0.0859; R^2^ = 0.9914), and the TPC was quantified as mg gallic acid equivalents (GAEs) per milliliter of the ethanolic propolis extract.

For the total flavonoid content (TFC) in the propolis samples, the aluminum chloride colorimetric assay was employed [20]. Propolis samples (100 μL) were mixed with 2% AlCl_3_ (100 μL), and, after homogenization and dark incubation (30 min), the absorbance at 420 nm was quantified. The calibration curve was established using a quercetin standard (10–60 µg/mL; y = 0.0093x − 0.0239; R^2^ = 0.9955), and the TFC was expressed as mg quercetin equivalents (QEs) per milliliter of the ethanolic propolis extract. All measurements were performed in triplicate (*n* = 3).

### 2.6. Antioxidant Capacity Assay

The antioxidant capacity was assessed using 1,1-Diphenyl-2-picryl-hydrazyl (DPPH) [21], 2,2-azino-bis-3-ethylbenzothiazoline-6-sulfonic acid (ABTS) [22], and ferric reducing antioxidant power (FRAP) [23]. For each measurement, 50 μL of the propolis extract was mixed with 150 μL of the appropriate solution (DPPH, ABTS, or FRAP). The absorbance was measured after 30 min of incubation. The calibration curves for the tests were established using a Trolox standard at 517 nm for the DPPH test, 732 nm for the ABTS test, and 593 nm for the FRAP test. The calibration equations were as follows: for DPPH, 30–70 µg/mL (y = −0.0108x + 0.8178; R^2^ = 0.9990); for ABTS, 8–60 ug/mL (y = −0.0103x + 0.8091; R^2^ = 0.9933); and for FRAP, 6–20 ug/mL (y = 0.0274x + 0.031; R^2^ = 0.9940). The antioxidant capacity was expressed as mg Trolox equivalents (TEs) per milliliter of the ethanolic propolis extract. Each measurement was repeated three times.

### 2.7. HPLC–MS/MS

The phenolic profile, including both identification and quantification, was determined following the methodology previously described [20]. The CE and OE, both before and after digestion, were analyzed using an ultra-high-pressure liquid chromatograph (Ekspert Ultra LC 100-XL system, Eksigent Technologies, Dublin, CA, USA) coupled to a triple quadrupole mass spectrometer operating in positive electrospray ionization (ESI) mode (AB Sciex Triple Quad 4500, Framingham, MA, USA). Chromatographic separation was performed on a LiChrospher 100 RP-18 endcapped column (125 mm × 4 mm i.d., 5 µm particle size) (Merck, Darmstadt, Germany) maintained at 30 °C, using a mobile phase composed of 0.1% formic acid and methanol in gradient mode, with a flow rate of 0.5 mL min^−1^. The gradient program was as follows: 0–1 min, 15% B; 1–17 min, 15–100% B; 17–21 min, 100% B; 21–22 min, 100–15% B; and 22–25 min, 15% B. The LC–MS/MS system was controlled using Analyst 1.6.2 software, and data processing was performed with Multiquant 3.0. For the quantitative analysis of phenolic compounds, commercial standards obtained from Sigma-Aldrich (Sigma-Aldrich, St. Louis, MO, USA) were employed. The analysis utilized a primary transition for quantification and a secondary transition for identification. Results were expressed as mean values ± standard deviation (SD) based on triplicate measurements.

### 2.8. Antibacterial Assay

The bacterial strains used in the antimicrobial assays were *Listeria monocytogenes* BM-01-02 1 (serotype 1/2a), BM-14 (serotype 1/2b), and BM-01-02 4 (serotype 4b) and *Salmonella enterica* strains FA0519 (ser. Typhimurium), FA0208 (ser. Infantis), and FA0032 (ser. Enteritidis). *Staphylococcus aureus* strain ATCC 25923 was also included in the assays.

Antibacterial activity was evaluated by measuring the inhibition zone diameter on Mueller–Hinton agar plates using the Clinical and Laboratory Standards Institute (CLSI) method [24]. Bacterial suspensions were standardized to a concentration of 0.5 McFarland standard (1.5 × 10^8^ CFU/mL, Becton Dickinson Company, Franklin Lakes, NJ, USA). An aliquot from each suspension was used for mass seeding on Petri dishes, and wells with a 6 mm diameter were prepared. In each well, 100 µL of propolis extract was added with a concentration of 200 mg/mL. The plates were incubated for 24 h at 37 °C. The assays were conducted in triplicate.

For the minimum bactericidal concentration (MBC) assay, bacterial suspensions were prepared at a concentration of 0.5 McFarland standard. A volume of 150 µL of the extract was added to 150 µL of Mueller–Hinton broth in a 96-well microplate for 1:2 serial dilutions, then 50 µL of the bacterial suspension was added to each well. Microplates were incubated for 24 h at 37 °C. From each well, 4.0 µL was plated on Mueller–Hinton agar plates and incubated for 24 h at 37 °C. The MBC was considered to be the lowest concentration that inhibited growth. The controls for both tests were Streptomycin (1 mg/mL) and ethanol 80% (*v*/*v*).

### 2.9. In Vitro Digestion and Relative Bioaccessibility

To simulate the oral, gastric, and intestinal phases of digestion, we conducted assays based on the protocol previously described by INFOGEST [25]. During the oral phase simulation, 1.5 mL of propolis extracts was mixed with simulated digestion fluids and the corresponding enzyme for 2 min at 37 °C. The gastric and intestinal phase simulations used the sample from the abovementioned oral phase, incubating for 2 h at 37 °C with continuous mixing. Each phase included a control (simulated digestion fluids without sample). All measurements were carried out in triplicate.

To estimate bioaccessibility, the total bioactive compounds and functional properties were measured in samples before and after in vitro digestion. The relative bioaccessibility (RB) percentages were calculated using the following formula previously described [26]:

$$RB\left(\%\right)=\frac{CD}{CI}\times 100$$

where CD represents the results after digestion (bioaccessible fraction) and CI represents the results before digestion.

### 2.10. Statistical Analysis

Data analysis was performed using Statgraphics Plus^®^ 5.1 software. Analysis of variance (ANOVA) was employed to assess the effect of treatments on dependent variables (i.e., extraction time on TPC). Significant differences between multiple means were determined with Tukey’s test, while paired mean comparisons were carried out using Student’s *t*-test.

## 3. Results and Discussion

### 3.1. Botanical Origin of Propolis

The analysis of propolis pollen grains obtained from the central zone of Chile indicates a multifloral origin (Table 2) with predominant species such as *Lithraea caustica* (28.2%), *Escallonia pulverulenta* (18.4%), and *Populus nigra* (13.6%). It is expected that propolis presents antioxidant and anti-inflammatory properties due to the high flavonoid and phenolic content of *L. caustica* [27]. Additionally, the *Populus* genus, to which *P. nigra* belongs, is one of the most documented botanical sources of propolis, particularly in temperate regions [28]. Their high content of phenolic acids and flavonoids enhances the antimicrobial efficacy of propolis [29,30]. Other important bioactive properties are expected due to other contributors, which include *Peumus boldus* (8.7%), *Cryptocarya alba* (7.8%), and *Eucalyptus* sp. (7.8%). *P. boldus* is widely recognized for its alkaloid and flavonoid composition, which could enhance the bioactivity of the propolis [31]. Similarly, *Eucalyptus* sp. is associated with antibacterial and expectorant properties, attributed to its high content of eucalyptol (1, 8-cineole) and terpenoids, which reinforces the potential use of this propolis in respiratory health formulations [32,33].

Analysis of other plant structures indicated that the high contribution of *L. caustica* trichomes (28.0%) supports the hypothesis that this species is a key contributor to the chemical profile of the evaluated propolis. The contribution of *Eucalyptus camaldulensis* vessels (18.0%) is also particularly relevant, as it has been widely documented for its antiseptic, anti-inflammatory, and wound-healing properties due to its phenolic-rich extracts [34]. These findings suggest that the botanical diversity of this propolis sample could enhance its pharmacological versatility, making it a potential candidate for therapeutic and nutraceutical applications.

### 3.2. Optimized Parameters for UAE Extraction Method

**Figure 1:**
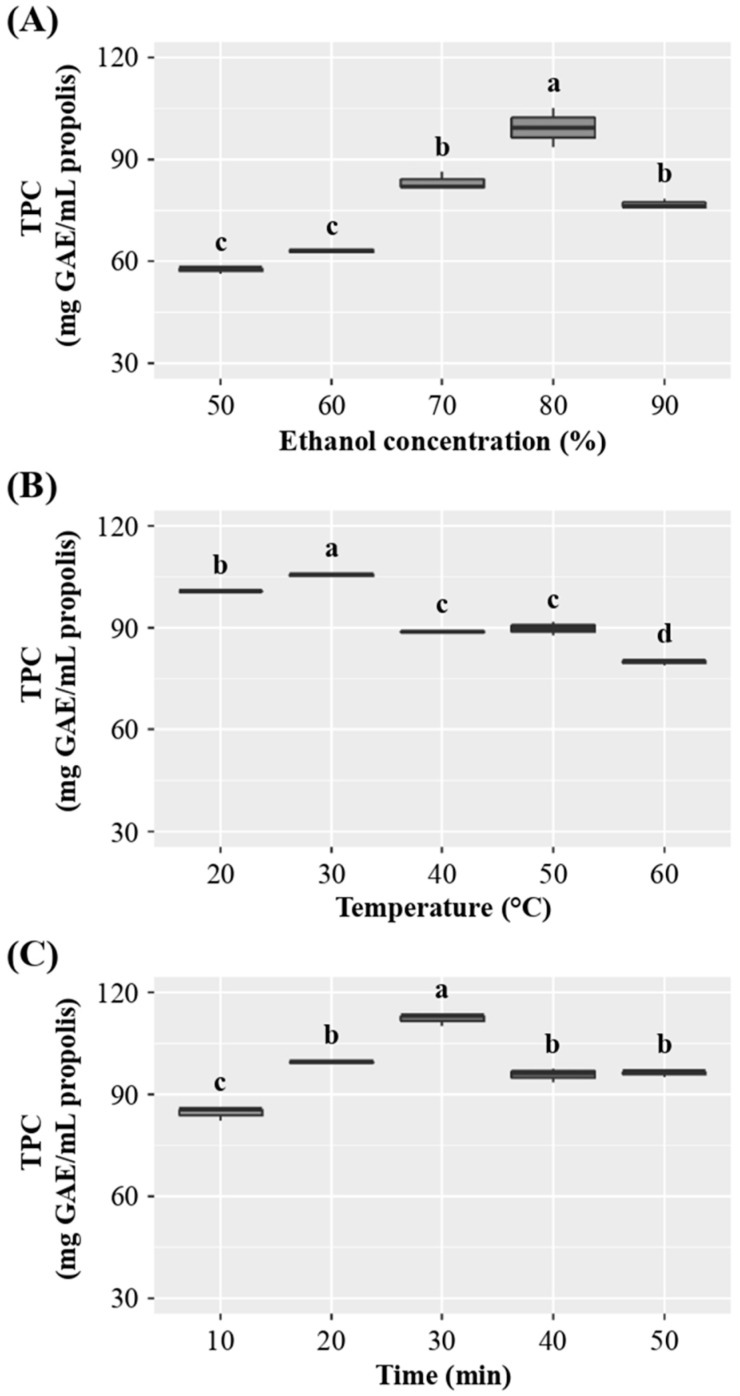
Extraction parameters impact on total polyphenol content (TPC) of ethanolic propolis extract. (**A**) Solvent concentration (% ethanol *v*/*v*), (**B**) Temperature (°C), and (**C**) Time (min). The effect of each extraction variable on TPC was assessed using ANOVA. Differences between means were determined with Tukey’s test, and means sharing the same letters indicate no significant differences (*p* < 0.05).

Beekeepers widely use ethanol to extract propolis due to its food-grade quality and effectiveness in extracting polyphenols from the matrix [35,36]. Therefore, we determined the TPC of the extracts obtained using various ethanol concentrations (50, 60, 70, 80, and 90%), temperatures (20, 30, 40, 50, and 60 °C), and times (10, 20, 30, 40, and 50 min) (Figure 1). All assays were conducted at 80 W, with a propolis/solvent ratio of 1:10 (*w*/*v*) g/mL. The maximum TPC (*p* < 0.05) was achieved with an ethanol concentration of 80% (*v*/*v*) (Figure 1A). The TPC increased progressively from 50 to 80% (*v*/*v*) ethanol concentration, whereas it significantly decreased from 80 to 90%. This behavior is consistent with findings previously reported [37,38]. Interestingly, the highest TPC at an 80% ethanol concentration has also been determined in propolis extracted by ultrasound at 100 W and 40 °C for 5 h [39].

The highest values of TPC were exhibited at an extraction temperature of 30 °C, whereas increasing temperatures (40, 50, and 60 °C) significantly decreased the TPC content (Figure 1B). By contrast, higher temperatures (59 °C) have been reported as optimal for Malaysian propolis [37]. In the case of propolis collected in Turkey, the optimal extraction temperature was 65 °C, using a 1:10 ratio, an ethanol concentration of 80%, and a 25-min ultrasound-assisted method [40].

An increase in TPC was observed with longer extraction times in the interval from 10 to 30 min, whereas the process conducted for 40 and 50 min yielded similar TPC as that conducted for 20 min (Figure 1C). The optimal extraction time of 30 min agrees with that reported in Italian propolis when a 1:10 (*w*/*v*) ratio of propolis/ethanol (70% *v*/*v*) was used [41].

The extraction of propolis using ethanol demonstrates optimal concentrations at 80% and an extraction time of 30 min, consistent with previous findings. However, the optimal temperature for this extraction was identified as 30 °C, which contrasts with reported parameters for propolis from other investigations that suggest higher temperatures.

### 3.3. Phytochemical Screening

According to previous reports, the chemical composition of propolis varies depending on the local geographical location and flora [2,42]. The central zone has a warm and subhumid Mediterranean-type climate, which allows the existence of native vegetation. Specifically, in the Mediterranean agroecological landscape, the predominant native vegetation that results is the renewal type of sclerophyll forest, characterized mainly by *Cryptocarya alba* (Chilean peumo), *Quillaja saponaria* Mol. (Quillay), and *Lithrea caustica* Mol. (Liters) species [43]. Consequently, evaluating a qualitative phytochemical screening provides an initial insight into the compounds present in Chilean propolis.

In the propolis obtained with both extraction methods, before and after in vitro digestion, we observed the presence of all the phytochemicals evaluated except for saponins (Table 3). The most abundant compounds corresponded to flavonoids and phenols, followed by coumarins and sugars. There were no differences in the intensity of color between the CE and OE before digestion, which exhibited an overall similar detected abundance of phytochemicals. Degradation of most determined compounds was observed after digestion, except for alkaloids and proteins, which exhibited higher stability during in vitro digestion (Table 3).

Similar results were found in roasted coffee beans, where tannins were not detected after the intestinal digestion stage [44]. This behavior can be explained by the formation of tannin–protein precipitates through hydrogen bonds and hydrophobic interactions during the oral phase [45]. It has also been reported that no intact proteins were visually detected in the soluble or insoluble fractions once the intestinal phase was completed, either in isolated proteins or in their food matrix [46]. This phenomenon might be the result of the action of enzymes such as pepsin and pancreatin, which can convert proteins into simpler peptide structures [47]. In the case of alkaloids, these compounds have also shown high stability in their bioaccessibility in hydroethanolic extracts of herbs, exhibiting no significant concentration decrease after in vitro intestinal digestion [48]. These findings emphasize the significance of extraction methods and digestive stability in assessing the bioactive potential of propolis.

### 3.4. Total Polyphenols, Flavonoid Content, and HPLC–MS/MS

Over 500 different metabolites have been identified in propolis, with polyphenols being the predominant compounds [49]. The TPC of the OE was 24.4 ± 0.4 mg GAE/mL, which is comparable to other Chilean propolis with values greater than 20.0 mg GAE/mL [9]. The optimization process for obtaining the ethanolic propolis extract resulted in a significant increase in TPC (23.4%) and TFC (45.2%) (Table 4). Likewise, ultrasound-assisted extraction in Romanian propolis with 70% ethanol, a 1:50 (*w*/*v*) ratio, and 15 min increased TPC by 7.2%, as compared to maceration [38]. In Thai propolis, ultrasound extraction also increased the TFC by 6.0% at 25 °C with 70% ethanol and a 1:10 (*w*/*v*) ratio [50]. These results indicate that ultrasound extraction improves both the yield of bioactive compounds and the efficiency of the extraction process (i.e., lower extraction temperature and time). These characteristics contribute to the environmental sustainability of the overall process [51]. Ultrasound waves produce a cavitation effect that facilitates the breakdown of cell walls, thereby promoting the release of phenolics and flavonoids into solvent [52]. As a result, ultrasound-assisted propolis extraction proves to be a better method than conventional extraction techniques, such as maceration.

The TPC relative bioaccessibility (RB) was 20.3% for the CE and 20.1% for the OE, whereas the RB of the TFC in the CE and OE was 7.0% and 7.6%, respectively (Table 4). During in vitro digestion, TPC experiences molecular modifications and degradation by digestive enzymes under acidic and alkaline conditions [53]. A similar behavior can be observed when analyzing the concentrations of certain model compounds as determined by chromatography. For both polyphenolic acids (e.g., chlorogenic acid) and flavonoids (e.g., rutin), a decrease in the available amount after digestion can be observed, regardless of the extraction method. Research has suggested that the intestinal phase may break down phenolic compounds like flavan-3-ol through processes such as autooxidation, polymerization, or complex formation in alkaline conditions [54]. Furthermore, bonds can break during digestion, leaving less stable free phenols that can form complex phenolic derivatives that spectrophotometric methods are unable to detect [55].

In conclusion, although the optimized extraction process significantly increased TPC and TFC in propolis, the relative bioaccessibility during in vitro digestion remained similar across methods. This indicates that molecular modifications and degradation in phenolic compounds occur uniformly, affecting bioaccessibility regardless of the extraction technique.

### 3.5. Antioxidant Capacity

The antioxidant capacity was assessed in the CE and OE, before and after simulated digestion, using DPPH, ABTS, and FRAP. The antioxidant capacity was significantly lower in the CE than in the OE (*p* < 0.05) before digestion (Table 4). The UAE resulted in increases of 4.5%, 39.5%, and 29.3% in DPPH, ABTS, and FRAP values, respectively. A similar trend was observed in Chinese propolis, where optimal UAE conditions led to a 95.5% increase in DPPH values and a 65.5% increase in ABTS values compared to conventional extraction methods [56]. Additionally, Thai propolis also exhibited an 18.5% increase in DPPH values under UAE conditions [51].

After digestion, the RB of the antioxidant capacity in terms of DPPH, ABTS, and FRAP was 56.6%, 62.5%, and 64.6% for the CE and 69.5%, 60.5%, and 61.9% for the OE, respectively (Table 4). By contrast, studies on Turkish propolis reported lower bioaccessibility values for ABTS (10.9%) and FRAP (5.6%) [57]. Additionally, research on Mexican propolis indicated substantial reductions of 98.1% in DPPH and 77.8% in ABTS during in vitro digestion, alongside an 80% loss of total polyphenol content (TPC) and a 90% loss of total flavonoid content (TFC) [58]. Our findings align with those of Wanyo et al. [59], who reported reductions of 43.0% and 24.0% in DPPH and FRAP values, respectively, in crude extracts of Purple Rice Bran subjected to the intestinal phase. Collectively, in vitro digestion studies have revealed a relevant variability in antioxidant stability and bioaccessibility across different propolis sources and extraction methods.

**Figure 2:**
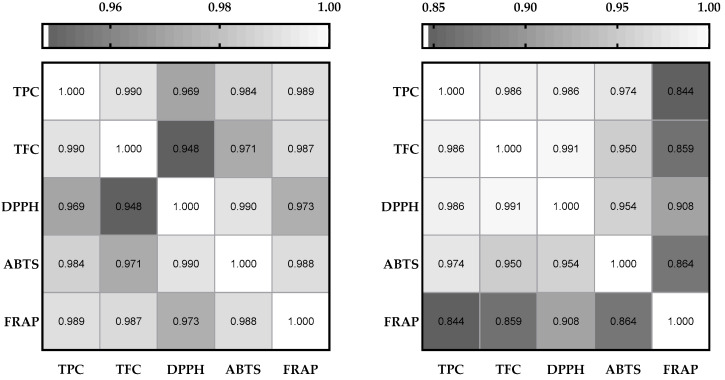
Pearson correlation heatmaps between total phenolic content (TPC), total flavonoid content (TFC), and antioxidant activity (DPPH, ABTS, FRAP) in ethanolic propolis extracts before (**left**) and after (**right**) simulated digestion (*n* = 6). All correlations shown are statistically significant (*p* < 0.05). The color scale represents the strength of the correlation coefficient (r).

In addition to the observed differences in antioxidant capacity and its retention following digestion, correlation analysis provided further insight into the relationship between bioactive compound content and functional performance (Figure 2). Statistically significant associations between TPC, TFC, and antioxidant capacity were consistently observed both before and after digestion. The analysis was performed using all replicates from both extraction methods (CE and OE), offering a broader perspective on compound–activity relationships. These findings indicate that, despite the partial degradation of phenolic and flavonoid compounds, their contribution to antioxidant activity is largely retained in the bioaccessible fraction. This underscores the relevance of UAE not only for enhancing the initial concentration of bioactives but also for preserving their functional efficacy under simulated gastrointestinal conditions.

### 3.6. Antibacterial Activity

Ethanolic extracts of propolis are promising sources of bioactive compounds with potential antibacterial properties, especially against Gram-positive bacteria [60]. The OE and CE showed, in general, higher antimicrobial activity against the *L. monocytogenes* strains as compared to the *S. enterica* strains (Table 5). These differences have also been reported in propolis extract obtained with an ethanol concentration of 80% (*v*/*v*) from Eastern Algeria, which exhibited greater inhibition of Gram-positive bacteria (*Staphylococcus aureus* and *Streptococcus agalactiae*) compared to Gram-negative bacteria (*Klebsiella pneumoniae*, *Escherichia coli*, and *Pseudomonas aeruginosa*) [61]. This variation can be explained by the fact that Gram-negative bacteria possess a complex cell wall that includes an outer membrane and other components [62]. Additionally, it has been observed that Gram-negative bacteria have efflux pumps that prevent the accumulation of propolis compounds, thereby contributing to their relatively higher tolerance [63].

The inhibition diameter results indicated no significant differences between the OE and CE for all evaluated bacteria (Table 5). However, the MBC of the OE for the *L. monocytogenes* strains and for *S. aureus* was 3.1 mg/mL, which was significantly lower than that found for the CE (6.3 mg/mL) (Table 5). Overall, the ultrasound extraction method for 30 min enhanced bioactivity against both *S. aureus* and *L. monocytogenes* compared to the CE, which could be related to the increase in phenolic acids and rutin in the OE.

Although the tested ethanol concentrations showed a low inhibition zone diameter, ethanol at 20% (*v*/*v*) was the MBC against all evaluated strains (Table 5). The ethanolic propolis extract concentration of 50 mg/mL corresponded to an ethanol concentration of 20% (*v*/*v*); therefore, the observed antimicrobial activity against Gram-negative bacteria could not be attributed uniquely to the effect of propolis. By contrast, the ethanol concentrations in the propolis dilutions controlling the Gram-positive bacteria were 1.3 and 2.5% (*v*/*v*) for the MBC of 3.1 and 6.3 mg/mL, respectively.

After the simulated digestion, both the OE and CE completely lost their antimicrobial activity against the tested bacteria, according to the inhibition zone and the MBC assays (Table 5). This loss may be attributed to a reduction in phytochemical diversity and concentration, as well as to the structural degradation or transformation of active compounds during gastrointestinal processing. Polyphenols, in particular, can be metabolized into derivatives with reduced or no bioactivity or may be rapidly degraded and eliminated. Furthermore, interindividual differences in gut microbiota composition can influence the metabolic fate of phenolic compounds, potentially altering their biological activity in vivo [64]. As this study was conducted using an in vitro digestion model, such variability could not be accounted for, which represents a limitation when extrapolating these results to physiological conditions.

## 4. Conclusions

Under the established optimal conditions, ultrasound-assisted extraction was shown to be an efficient technique for obtaining ethanolic extracts from propolis. This method yielded extracts with enhanced bioactive compound concentrations and biological activity compared to conventional maceration-based extraction. Although the simulated digestion process resulted in a decrease in total polyphenol and flavonoid content and in antioxidant capacity, these properties remained higher in the extracts with the optimized ultrasound system. Overall, this study highlights the importance of developing optimized protocols for novel extraction techniques to generate propolis extracts with superior bioactive attributes. Future research may also benefit from integrating complementary analyses, such as solid content quantification, to strengthen comparisons across different extraction formats.

## Figures and Tables

**Table 1:** Phytochemical screening tests conducted to characterize the ethanolic propolis extracts.

Phytochemical	Test	Positive Result
Tannins ^a^	500 μL extract + 500 μL H_2_O + 1–2 drops of FeCl_3_ (10%)	Blue-Green color precipitate
Flavonoids ^b^	1 mL extract + 2 mL of 2% NaOH solution+ drops diluted HCl	Colorless
Coumarins ^c^	1 mL extract + 10% NaOH + Chloroform	Yellow color
Alkaloids ^d^	2 mL extract + drops of Dragendorff’s reagents	Reddish-brown precipitate
Phenols ^e^	Extract + drops of K_2_Cr_2_O_7_	Dark color
Proteins ^f^	2 mL extract + 1 drop of CuSO_4_ (2%) + 1 mL of Ethanol (95%) + 1 pellet KOH	Pink color
Sugars ^g^	1 mL extract + 1 mL Fehling’s solution A+ 1 mL Fehling’s solution B + boiled in water bath	Red precipitate
Saponins ^h^	1 mL extract + 2 mL water (vigorously shaken)	Foam for ten minutes

The positive controls were ^a^ tannic acid, ^b^ catechin, ^c^ coumarin, ^d^ oxymatrine, ^e^ gallic acid, ^f^ casein, ^g^ sucrose, ^h^ oats. The negative control was distilled water.

**Table 2:** Botanical origin of propolis from the central zone of Chile.

Botanical Attributes		Plant Source	Contribution (%)
Pollen		*Cryptocarya alba*	7.8
		*Peumus boldus*	8.7
		*Escallonia pulverulenta*	18.4
		*Melilotus indicus*	4.9
		*Quillaja saponaria*	2.9
		*Populus nigra*	13.6
		*Schinus latifolius*	7.8
		*Eucalyptus* sp.	7.8
		*Lithraea caustica*	28.2
Plant structures	Trichomes	*Lithraea caustica*	28.0
		*Retanilla trinervia*	12.0
	Vessels	*Populus nigra*	16.0
		*Eucalyptus camaldulensis*	18.0
		*Nothofagus obliqua*	20.0
	Epidermis	*Carduus picnocephalus*	6.0

**Table 3:** Qualitative phytochemical screening of propolis samples before and after in vitro digestion.

Phytochemical	Negative Control	Positive Control	Before Digestion		After Digestion	
			Conventional Extract	Optimized Extract	Conventional Extract	Optimized Extract
Tannins	-	+	+	+	-	-
Flavonoids	-	+	+++	+++	++	++
Phenols	-	+	+++	+++	++	++
Coumarins	-	+	++	++	+	+
Alkaloids	-	+	+	+	+	+
Proteins	-	+	+	+	+	+
Sugars	-	+	++	++	+	+
Saponins	-	+	-	-	-	-

+++ highly abundant, ++ moderately present, + present, - absent.

**Table 4:** Phenolic content and antioxidant capacity of ethanolic propolis extract obtained with conventional and optimized method, before and after simulated digestion.

Analysis		Before Digestion		After Digestion		Bioaccessibility (%)	
		CE	OE	CE	OE	CE	OE
Phenolic compounds	TPC (mg GAE/mL)	18.7 ± 0.1 ^b^	24.4 ± 0.4 ^a^	3.8 ± 0.1 ^d^	4.9 ± 0.0 ^c^	20.3	20.1
	TFC (mg QE/mL)	8.6 ± 0.4 ^b^	15.7 ± 0.7 ^a^	0.6 ± 0.0 ^d^	1.2 ± 0.1 ^c^	7.0	7.6
Antioxidant Capacity (mg TE/mL)	DPPH	60.1 ± 0.3 ^b^	62.9 ± 0.3 ^a^	34.0 ± 0.7 ^d^	43.7 ± 0.4 ^c^	56.6	69.5
	ABTS	21.6 ± 0.6 ^b^	35.7 ± 1.0 ^a^	13.5 ± 0.3 ^c^	21.6 ± 1.4 ^b^	62.5	60.5
	FRAP	36.2 ± 1.2 ^b^	51.2 ± 0.1 ^a^	23.4 ± 3.3 ^c^	31.7 ± 0.5 ^b^	64.6	61.9
HPLC–MS/MS (ppb)	Cinnamic acid	1900 ± 100 ^b^	3500 ± 100 ^a^	nd	500 ± 10 ^c^	-	14.3
	Ferulic acid	4835 ± 400 ^b^	8350 ± 600 ^a^	695 ± 40 ^c^	790 ± 60 ^c^	14.4	9.5
	Chlorogenic acid	78.0 ± 3.5 ^b^	168.5 ± 0.5 ^a^	-	-	-	-
	Caffeic acid	73.0 ± 2.0 ^b^	88.0± 1.5 ^a^	-	-	-	-
	Coumaric acid	51.0 ± 0.5 ^d^	77.5 ± 1.5 ^c^	94.5 ± 1.5 ^b^	123.0 ± 1.5 ^a^	185.3	158.7
	Rutin	86.5 ± 2.5 ^b^	212.0 ± 4.5 ^a^	3.0 ± 0.5 ^c^	4.5 ± 1.1 ^c^	3.5	2.1

Results are shown as mean ± standard deviation (*n* = 3). The effect of the extraction method and digestion process on the extract properties was assessed using ANOVA. Differences between means were determined with Tukey’s test, and means sharing the same letters within each row indicate no significant differences (*p* < 0.05). OE: optimal ethanolic UAE extract; CE: conventional ethanolic extract; TPC: total polyphenol content; TFC: total flavonoid content; DPPH: 1,1-Diphenyl-2-picryl-hydrazyl; ABTS: 2,2-azino-bis-3-ethylbenzothiazoline-6-sulfonic acid; FRAP: ferric reducing antioxidant power; -: not detected.

**Table 5:** Antibacterial capacity of the ethanolic propolis extract quantified through inhibition zone diameter (mm) and minimum bactericidal concentration (MBC) (mg/mL) before and after simulated digestion.

Assay	Bacteria	Before Digestion		After Digestion		Streptomycin	Ethanol
		Conventional Extract	Optimized Extract	Conventional Extract	Optimized Extract		
Inhibition diameter (mm)	*S. enterica* ser. Infantis	7.7 ± 0.5	7.0 ± 0.0 ^ns^	-	-	23.0 ± 0.5	7.0 ± 0.0
	*S. enterica* ser. Enteritidis	8.0 ± 0.8	9.0 ± 0.0 ^ns^	-	-	24.0 ± 0.5	7.7 ± 0.5
	*S. enterica* ser. Typhimurium	8.3 ± 0.5	9.0 ± 0.0 ^ns^	-	-	27.0 ± 0.9	7.0 ± 0.0
	*L. monocytogenes* ser. 1/2a	15.3 ± 0.5	15.3 ± 0.5 ^ns^	-	-	27.0 ± 0.8	8.7 ± 0.5
	*L. monocytogenes* ser. 1/2 b	14.0 ± 0.0	16.5 ± 0.4 *	-	-	29.0 ± 0.0	8.7 ± 0.5
	*L. monocytogenes* ser. 4b	14.0 ± 1.6	16.0 ± 0.0 ^ns^	-	-	28.0 ± 0.5	9.0 ± 0.0
	*S. aureus*	24.7 ± 1.9	23.0 ± 2.2 ^ns^	-	-	28.0 ± 0.0	7.0 ± 0.0
MBC (mg/mL)	*S. enterica* ser. Infantis	50.0 ± 0.0	50.0 ± 0.0 ^ns^	-	-	1.0 ± 0.0	20.0 ± 0.0 ^a^
	*S. enterica* ser. Enteritidis	50.0 ± 0.0	50.0 ± 0.0 ^ns^	-	-	0.3 ± 0.0	20.0 ± 0.0 ^a^
	*S. enterica* ser. Typhimurium	50.0 ± 0.0	50.0 ± 0.0 ^ns^	-	-	0.5 ± 0.0	20.0 ± 0.0 ^a^
	*L. monocytogenes* ser. 1/2a	6.3 ± 0.0	3.1 ± 0.0 ***	-	-	0.03 ± 0.0	20.0 ± 0.0 ^a^
	*L. monocytogenes* ser. 1/2 b	6.3 ± 0.0	3.1 ± 0.0 ***	-	-	0.02 ± 0.0	20.0 ± 0.0 ^a^
	*L. monocytogenes* ser. 4b	6.3 ± 0.0	3.1 ± 0.0 ***	-	-	0.03 ± 0.0	20.0 ± 0.0 ^a^
	*S. aureus*	6.3 ± 0.0	3.1 ± 0.0 ***	-	-	0.03 ± 0.0	20.0 ± 0.0 ^a^

Assays results are shown as mean ± standard deviation (*n* = 3). The activity of conventional and optimized extracts, for each bacterium and parameter, was compared using Student’s *t*-test (ns = not significant *p* ≥ 0.05, * = *p* < 0.05, *** = *p* < 0.001). -: no activity. ^a^ MBC in ethanol% (*v*/*v*).

## Data Availability

Data will be made available on request.
